# Systematic review of economic evaluation studies in psychiatric pharmacogenomics

**DOI:** 10.1192/j.eurpsy.2021.1024

**Published:** 2021-08-13

**Authors:** C. Mitropoulou, K. Karamperis, M. Koromina, P. Papantoniou, M. Skokou, F. Kanellakis, K. Mitropoulos, A. Vozikis

**Affiliations:** 1 Research And Development, The Golden Helix Foundation, London, United Kingdom; 2 Pharmacy, University of Patras, Patras, Greece; 3 Economics, Warwick School of Business, London, United Kingdom; 4 Medical School, University of Athens, Athens, Greece; 5 Economics, University of Piraeus, Piraeus, Greece

**Keywords:** Pharmacogenomics, Cost-effectiveness analysis, cost analysis, Systematic review

## Abstract

**Introduction:**

Nowadays, many relevant gene-drug associations have been discovered, but pharmacogenomics (PGx)-guided treatment needs to be cost-effective as well as clinically beneficial to be incorporated into standard health-care.

**Objectives:**

To address current challenges, this systematic review provides an update regarding previously published studies, which assessed the cost-effectiveness of pharmacogenomics testing for the prescription of antidepressants and antipsychotics.

**Methods:**

Our initial screening revealed 1159 articles, which was subsequently reduced to 32 articles, deducted by analysis of their abstract. Full-text analysis performed by all authors resulted in 18 papers that were further included in the analysis.

**Results:**

Of the 18 studies evaluations, 16 studies (88.89%) drew conclusions in favor of PGx testing, of which 9 (50%) were cost-effective and 7 (38.9%) were less costly based on cost analysis. In brief, we found sufficient evidence on the cost-effectiveness of PGx in psychiatric disease care. More precisely, supportive evidence exists for CYP2D6 and CYP2C19 gene-drug associations and for combinatorial PGx panels, but evidence is limited for many other drug–gene combinations. Amongst the limitations of the field are the unclear explanation of perspective and cost inputs in many economic studies, as well as the underreporting of study design elements, which can influence significantly the economic evaluations.

**Conclusions:**

Overall, this systematic review highlights the need for additional research on economic evaluations of PGx implementation with an emphasis on psychiatric pharmacogenomics.

